# Steroid Refractory and Plasma Exchange Partially Responsive Longitudinally Extensive Transverse Myelitis Due to Tumor Necrosis Factor-Alpha (Etanercept): A Case Report

**DOI:** 10.3390/clinpract16050086

**Published:** 2026-04-29

**Authors:** Jelena Stojsavljevic, Rafael R. Perez, Emilia Petcu, Celestine Odenigbo, Cristian Madrid, Igor Dumic, Charles W. Nordstrom

**Affiliations:** 1 Department of Hospital Medicine, Mayo Clinic Health System, Eau Claire, WI 54703, USA; stojsavljevic.jelena@mayo.edu (J.S.); petcu.emilia@mayo.edu (E.P.); odenigbo.celestine@mayo.edu (C.O.); madrid.cristian@mayo.edu (C.M.); nordstrom.cw@mayo.edu (C.W.N.); 2 Mayo Clinic College of Medicine and Science, Rochester, MN 55905, USA; perezrodriguez.rafael@mayo.edu; 3 Department of Neurology, Mayo Clinic Health System, Eau Claire, WI 54703, USA

**Keywords:** transverse myelitis, longitudinally extensive transverse myelitis, multiple sclerosis, biologic therapy, TNF-alpha

## Abstract

**Background:** Acute transverse myelitis (ATM) is an inflammatory disorder of the spinal cord with heterogeneous etiologies, including autoimmune, infectious, paraneoplastic, and drug-induced causes. Tumor necrosis factor-alpha (TNF-α) inhibitors have been infrequently associated with inflammatory central nervous system events, including transverse myelitis. TNF-inhibitor-associated myelitis typically presents with short-segment lesions, a normal brain MRI, and partial responsiveness to corticosteroids. Longitudinally extensive transverse myelitis (LETM) and steroid-refractory cases are uncommon. **Case Presentation:** A 39-year-old woman with psoriatic arthritis treated with etanercept for two years presented with subacute progressive bilateral lower-extremity sensory loss and weakness. MRI revealed a T2 hyperintense spinal cord lesion extending from T11 to L1 with gadolinium enhancement, consistent with transverse myelitis, while brain MRI was normal. Cerebrospinal fluid analysis showed lymphocytic pleocytosis, elevated protein, oligoclonal bands, and increased kappa free light chains. Extensive infectious, metabolic, paraneoplastic, and autoimmune testing, including aquaporin-4 and MOG antibodies, was negative. Despite high-dose intravenous corticosteroids and the discontinuation of etanercept, the patient experienced clinical worsening with lesion expansion, meeting criteria for LETM, and developed urinary retention. She subsequently underwent plasma exchange, resulting in radiologic improvement and moderate clinical recovery. **Conclusions:** This case highlights an atypical presentation of TNF-inhibitor-associated myelitis characterized by a biphasic course, longitudinally extensive spinal cord involvement, steroid refractoriness, and responsiveness to plasma exchange. These features suggest either an unusually severe TNF-inhibitor-related inflammatory phenotype or a TNF-inhibitor-triggered antibody-mediated demyelinating process. Reports of TNF-inhibitor-associated myelitis evolving into longitudinally extensive, steroid-refractory disease remain limited, and this presentation may broaden the recognized clinical spectrum of TNF-α-related CNS inflammatory events. Close neurologic follow-up and heightened awareness of severe CNS complications associated with TNF-α inhibitors are warranted.

## 1. Background

Acute transverse myelitis (ATM) describes inflammation of the spinal cord involving part or all of the transverse section at any longitudinal level [1,2,3,4,5]. It is classified into two subtypes with clinical and diagnostic implications: short-segment transverse myelitis (involving <3 vertebral segments) and longitudinally extensive transverse myelitis (LETM) (extending over three or more contiguous vertebral segments on MRI) [1,2,3,4,5]. Short-segment transverse myelitis, which may be partial or complete depending on the degree of transverse involvement, is more commonly associated with multiple sclerosis (MS), idiopathic acute transverse myelitis, and post-infectious or post-vaccination myelitis [1,2]. LETM is typical for AQP4-IgG-seropositive neuromyelitis optica spectrum disorder (NMOSD) and is more frequently observed than short lesions in acute disseminated encephalomyelitis (ADEM), particularly in association with MOG antibody-associated disease (MOGAD). Long spinal cord lesions may also be seen in myelitis due to sarcoidosis and paraneoplastic syndromes [1,2,3,4,5].

Drug-induced ATM generally arises from immune-mediated mechanisms rather than direct toxicity [6]. Well-documented triggers include immune checkpoint inhibitors, tumor necrosis factor-alpha (TNF-α) inhibitors, tyrosine kinase inhibitors, chimeric antigen receptor T-cell (CAR T-cell) therapy, and several vaccines (hepatitis B, measles–mumps–rubella, and diphtheria–tetanus–pertussis) [7]. TNF-alpha inhibitors, widely used to treat rheumatoid arthritis (RA), psoriatic arthritis (PsA), and inflammatory bowel disease, have been linked to demyelinating and other inflammatory CNS disorders, including ATM, despite their immunosuppressive properties [6,7,8,9,10,11].

TNF-α-inhibitor-associated myelitis is a rare complication of anti-TNF-α treatment. In a recent review article, only 6 out of 122 (5%) patients with CNS demyelinating disorders related to anti-TNF-α were diagnosed with transverse myelitis (4). TNF-inhibitor-associated myelitis can present as either short-segment or longitudinally extensive lesions. Key distinguishing features include a temporal relationship to TNF inhibitor exposure (mean exposure duration 17.61 months in a recent review), typically normal brain MRI at presentation, negative AQP4-IgG and MOG-IgG antibodies, presence of CSF pleocytosis or oligoclonal bands, and gadolinium enhancement reflecting active inflammation in many cases [4].

## 2. Case Presentation

A 39-year-old woman with PsA, well controlled on etanercept for two years, developed progressive bilateral lower-extremity numbness, tingling, and weakness over the two weeks following her last etanercept dose. She initially noticed a painful, hypersensitive sensation in her right thigh that was intermittent but became persistent. Over the following week, the pain resolved; however, she developed numbness extending from the umbilicus to her right foot, along with new painful sensations in the left leg. She remained ambulatory but described *“not being able to feel the floor while walking.”* She reported having no back pain, urinary retention, constipation, diarrhea, recent trauma, toxic exposure, sick contacts, or tick bites.

Her medical history included hypertension treated with methyldopa, gastroesophageal reflux disease managed with pantoprazole, and anxiety, for which she reported occasional marijuana use. She was a lifelong nonsmoker and did not consume alcohol or other illicit drugs. General physical examination demonstrated intact cranial nerves and normal cardiovascular and pulmonary findings.

On neurological examination, the patient appeared in no acute distress. Mental status examination revealed that she was awake, alert, and oriented to self, place, and time; language function was preserved, and she followed commands appropriately. Cranial nerve examination demonstrated that pupils were equal and reactive to light; visual acuity was not formally assessed, and no visual field deficits were observed. Extraocular movements were intact without dysconjugate gaze. Facial movements were symmetrical, hearing was intact, and the uvula and tongue were midline. The motor examination revealed normal bulk and tone. Strength was full throughout except for mild weakness of the right lower extremity, with right hip flexion and knee extension, as well as foot dorsiflexion and plantar flexion, graded as Medical Research Council (MRC) 4/5 strength; all other muscle groups demonstrated MRC 5/5 strength. Sensory examination revealed a sensory level at approximately the umbilicus, with decreased pinprick and vibration sensation at and around this level. Deep tendon reflexes were 1+ throughout, with relatively brisk reflexes at the right patellar tendon (3+) and an extensor plantar response; no clonus was appreciated. Coordination testing showed no dysmetria. Gait was notable for a mildly right hemiparetic pattern.

**Figure 1:**
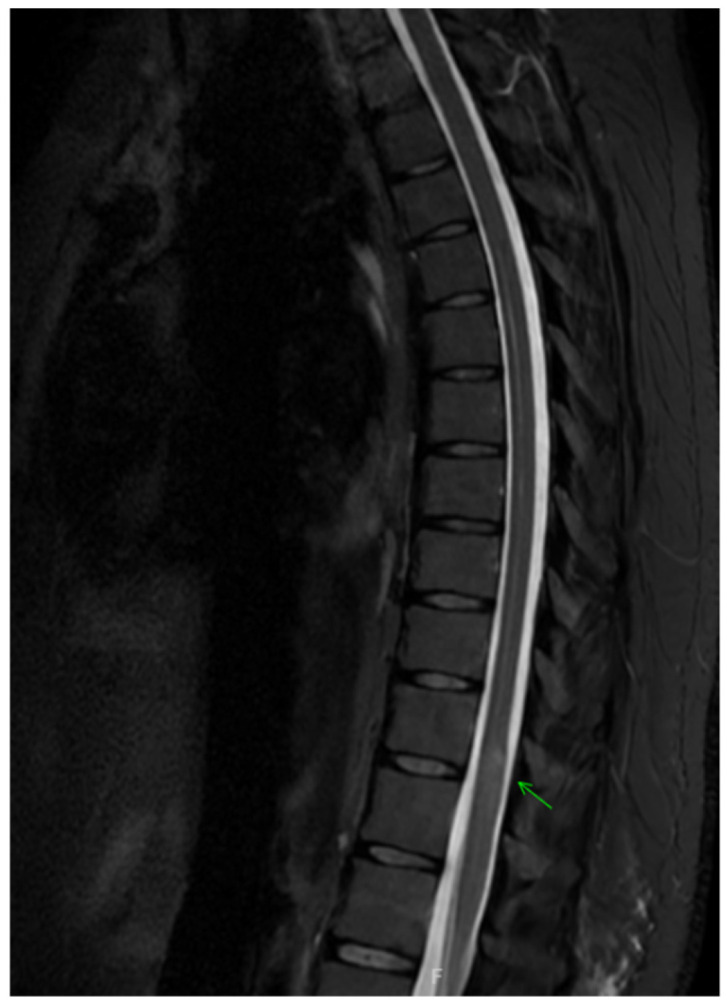
Axial T1 image demonstrating ill-defined T2 hyperintense spinal cord lesion extending from T11 to L1 ( arrow) consistent with transverse myelitis.

**Figure 2:**
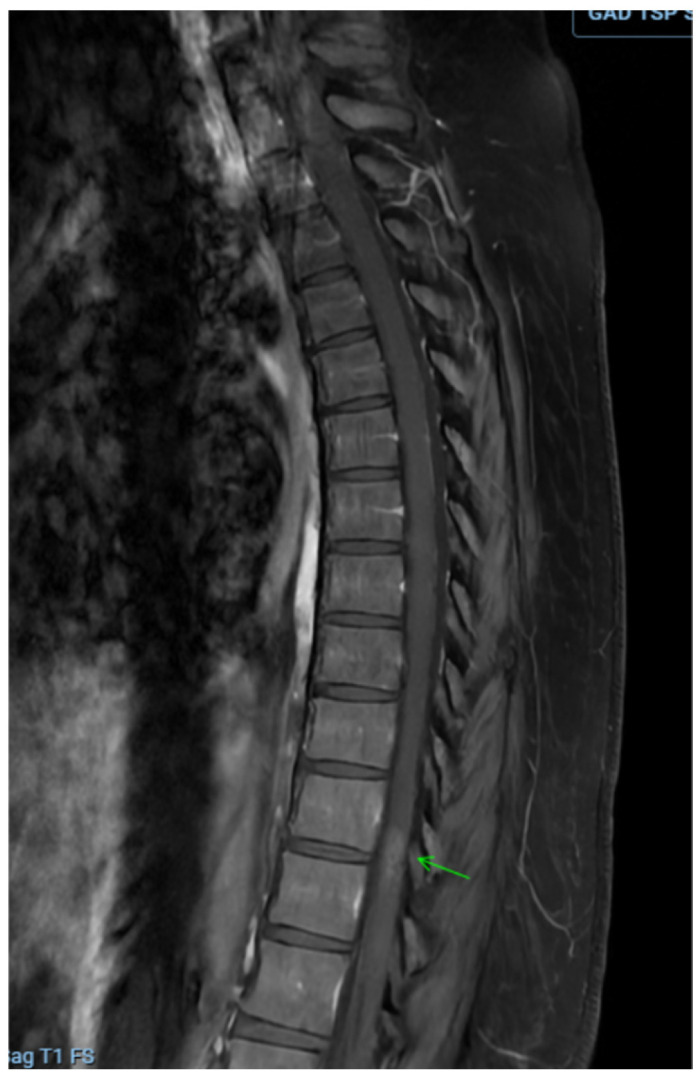
Sagittal image depicting contrast enhancement in T1 sequences.

Workup for suspected ATM included MRI of the brain, thoracic, and lumbar spine with and without contrast. Imaging showed an ill-defined T2 hyperintense spinal cord lesion extending from T11 to L1, consistent with transverse myelitis (Figure 1 and Figure 2). Brain MRI revealed no white-matter abnormalities, representing evidence against central nervous system demyelinating disorders such as MS, NMOSD, or MOGAD. In the context of ongoing TNF-inhibitor exposure, the leading diagnosis was etanercept-associated transverse myelitis.

Extensive laboratory testing was performed to exclude infectious, metabolic, paraneoplastic (which includes serum and the CSF Mayo Clinic paraneoplastic myelopathy panel including ANNA-1 (anti-Hu), ANNA-2, ANNA-3, amphiphysin-IgG, CRMP-5-IgG (anti-CV2), PCA-1 (anti-Yo), PCA-2, PCA-Tr (DNER), GAD65, GFAP-IgG, aquaporin-4-IgG (NMO-IgG), GABA-B antibody, and neurochondrin) and autoimmune causes. Renal and hepatic panels were normal. CRP was within normal limits, arguing against significant systemic inflammation. Vitamin B12 was low-normal at 356 ng/L (232–1245), with normal methylmalonic acid. Vitamin D was low at 15 ng/mL (20–80). Thiamine, folic acid, TSH, and copper were within normal limits. ANA was positive at 1:80 (homogeneous), but reflex testing for dsDNA, SS-A/Ro, SS-B/La, Sm, RNP, Scl-70, and Jo-1 antibodies was negative.

Lumbar puncture showed clear, colorless CSF with 19 nucleated cells/µL (0–5), predominantly lymphocytes (97%). Protein was mildly elevated at 49 mg/dL (15–45), and glucose was 54 mg/dL. Cytology was negative for malignancy. CSF kappa free light chains were elevated (>0.1 mg/dL), and oligoclonal banding identified four bands. Because of the high prevalence of tick-borne diseases in northwest Wisconsin, additional blood and CSF tests were obtained, and tick-borne panels returned negative. Both CSF and serum were tested on admission for autoimmune and CNS demyelinating disease panels, including NMO/AQP4 and MOG antibodies, prior to the initiation of any immunosuppressive therapy. The standard reference method is a live cell-based flow cytometric assay, which demonstrates >80% sensitivity and >99% specificity in serum. Serum NMO/AQP4 and MOG antibodies were assessed using a live cell-based FACS assay obtained on the second day of hospitalization, approximately two weeks after the onset of initial symptoms. Serum MOG-IgG testing using live cell-based assays with full-length human MOG is considered the gold standard. For evaluation of paraneoplastic neurological syndromes and autoimmune encephalitis, both serum and CSF were analyzed using a two-tiered approach: initial screening with a tissue-based indirect immunofluorescence assay (TIFA), followed by confirmatory, protein-specific testing based on whether the target antigen is intracellular or located on the cell surface. All testing was performed at Mayo Clinic Laboratories (Rochester, MN, USA) in accordance with standardized institutional protocols. Results from both CSF and serum analyses were negative.

After infectious causes of ATM were excluded, treatment was initiated with IV methylprednisolone 1000 mg daily for five days, resulting in only a minimal improvement in strength. Etanercept was discontinued, and the patient was counseled to avoid future TNF-inhibitor therapy. She participated in physical and occupational therapy and was discharged with plans for continued outpatient rehabilitation.

Fourteen days later, she was readmitted with worsening bilateral lower-extremity numbness and increased right-leg weakness. She was also found to have urinary retention. Repeat neurological examination showed that the upper extremity strength was preserved bilaterally, with all tested muscle groups demonstrating full strength (Medical Research Council [MRC] grade 5/5). In contrast, there was marked bilateral lower extremity weakness, with right greater than left: hip flexion (right MRC 2/5, left MRC 3/5), hip extension (right 3/5, left 4/5), hip adduction (right 3/5, left 3/5), hip abduction (right 4/5, left 5/5), knee extension (right 3/5, left 5/5), knee flexion (right 1/5, left 4/5), ankle dorsiflexion (right 1/5, left 5/5), and ankle plantarflexion (right 3/5, left 5/5). Deep tendon reflexes were reduced in the upper extremities (brachioradialis and biceps 1+; triceps trace bilaterally) and brisk in the lower extremities (patellar reflexes right 3+, left 2+; Achilles reflexes right 3+, left 2+). Hoffmann sign and ankle clonus were absent bilaterally; plantar responses were extensor bilaterally. Sensory examination revealed diminished light touch and pinprick sensation below the umbilicus, more pronounced on the right, with allodynia over the right thigh and absent vibration sense in both lower extremities; temperature and joint position sense were not assessed. Coordination testing demonstrated mild right-sided dysmetria on finger-to-nose and finger tracing, while heel-to-shin testing could not be performed. Gait assessment was deferred for patient safety.

**Figure 3:**
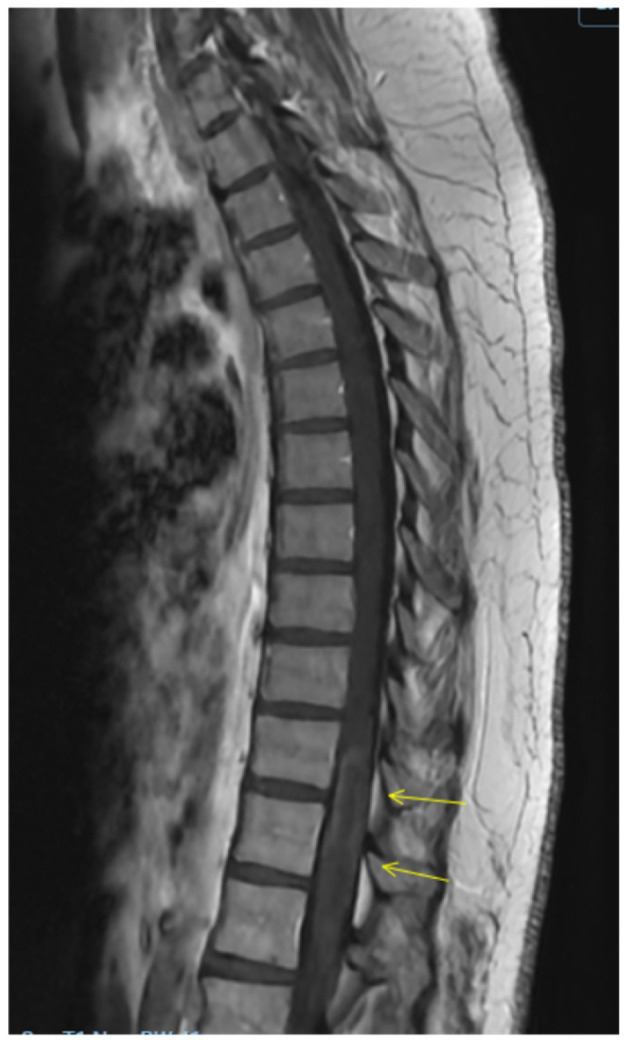
Sagittal T2 image demonstrating interval expansion of the T2 hyperintense contrast enhancement, now involving nearly the entire cord at T11–T12 and extending from T10 to L1, thereby meeting the criteria for a longitudinally extensive transverse myelitis.

**Figure 4:**
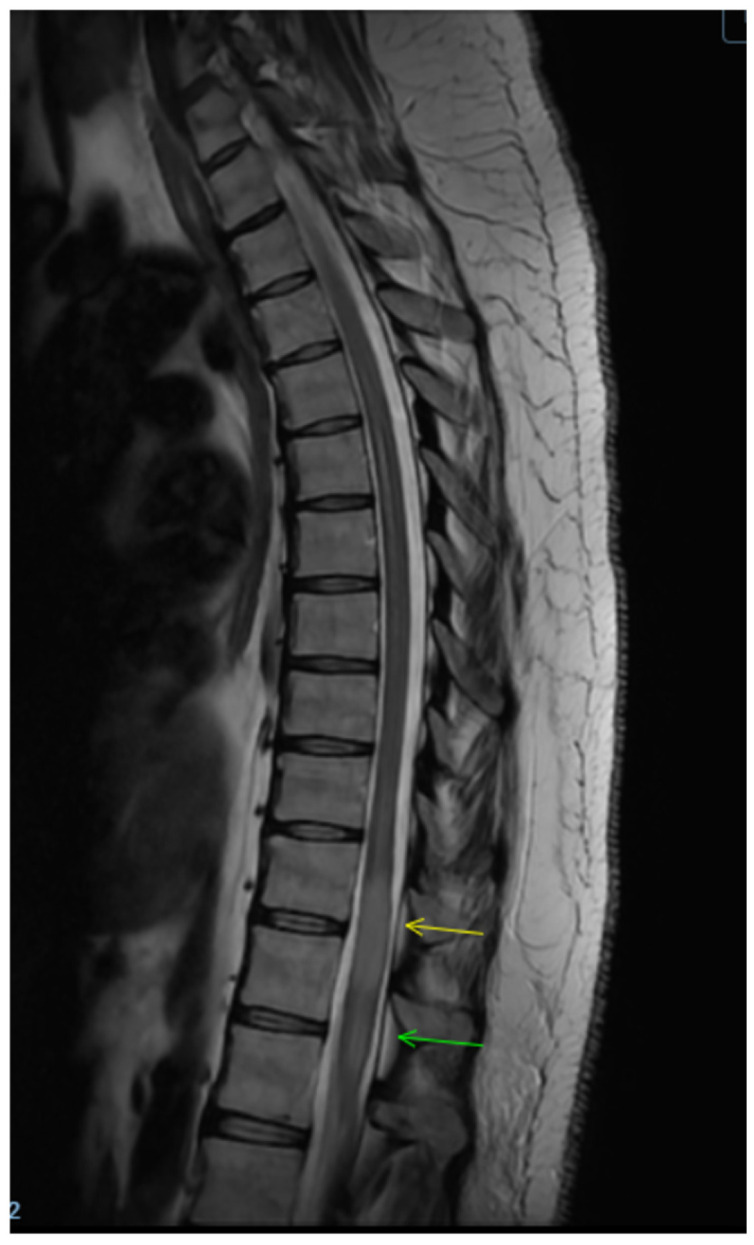
T2 axial image demonstrates interval expansion of the T2 hyperintense lesion, now extending from T10 to L1, thereby meeting the criteria for a longitudinally extensive transverse myelitis.

**Figure 5:**
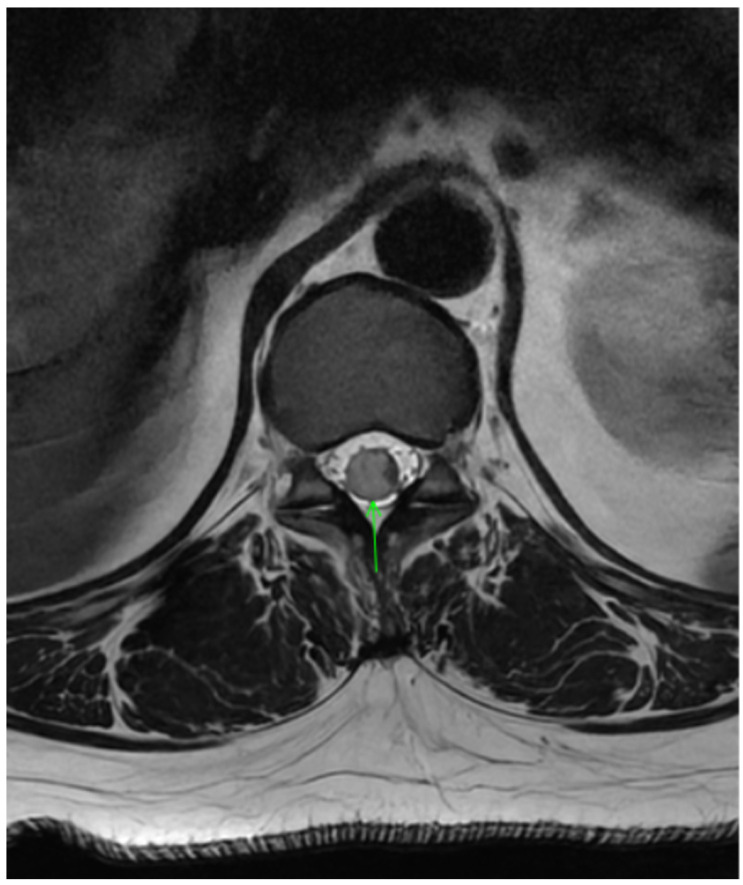
Thoracic MRI, T1 post-gadolinium obtained after PLEX demonstrated a decrease in lesion size, indicating partial radiologic response.

**Figure 6:**
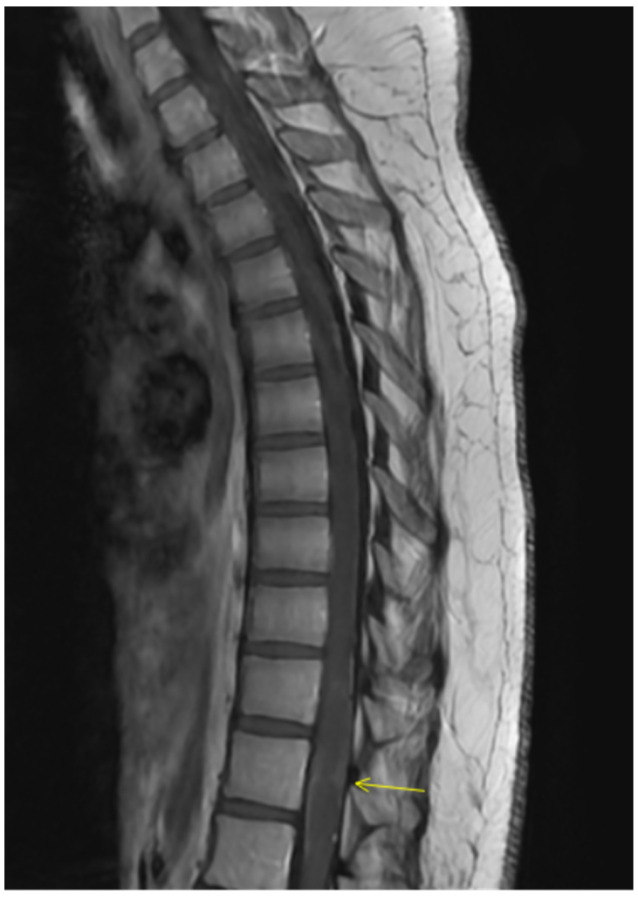
Image demonstrating a decrease in T2 contrast enhancement size, indicating partial radiologic response.

Repeat spine MRI demonstrated interval expansion of the T2 hyperintense lesion, now involving nearly the entire cord at T11–T12 and extending from T10 to L1, thereby meeting criteria for a longitudinally extensive transverse myelitis, without new cervical or thoracic lesions (Figure 3 and Figure 4). Optical coherence tomography showed no evidence of optic neuritis. Given recurrence and progression, plasma exchange (PLEX) was initiated, and she completed seven treatments over 14 days. Follow-up thoracic MRI after PLEX demonstrated a decrease in T2 lesion size, indicating partial radiologic response (Figure 5 and Figure 6). She continued intensive inpatient rehabilitation with gradual clinical improvement and was ultimately discharged for continued rehabilitation. Clinically, she experienced subjective improvement, most notably resolution of urinary retention, though she had not returned to her baseline strength. Outpatient neurological follow-up and serial imaging was recommended.

## 3. Discussion

Acute transverse myelitis is a devastating neurological syndrome marked by spinal cord inflammation. This inflammatory process damages the myelin sheath, producing acute bilateral and symmetric motor deficits, a well-defined sensory level, and varying degrees of autonomic dysfunction [1,2,3,4,5]. Its etiology is broad, including infectious, autoimmune (inflammatory), vascular, paraneoplastic, and post-vaccination causes [1,2,3,4]. Approximately 30% of cases remain idiopathic despite comprehensive evaluation [5]. ATM has an estimated incidence of 24.9 per million person-years, with substantial variation by underlying etiology [5]. Among identified causes, multiple sclerosis (MS) accounts for the largest proportion of cases, with nearly half of patients ultimately diagnosed with MS and about one-fifth presenting with ATM as their first MS event [5,6,7]. Antibody-mediated disorders, such as AQP4-IgG-positive neuromyelitis optica spectrum disorder (NMOSD) and MOG-IgG-associated disease, account for roughly half of previously idiopathic longitudinally extensive transverse myelitis (LETM) cases [6,7]. Infectious and para-infectious etiologies are less common in adults but represent up to 60% of pediatric ATM cases [1,2,3,4,5].

TNF-α inhibitor therapy is associated with a spectrum of adverse effects. The most common, generally mild, include infections (particularly upper respiratory tract infections and nasopharyngitis), injection-site reactions, and headache [12,13,14,15]. More serious complications, though less frequent, include severe infections (notably tuberculosis and opportunistic infections), malignancies (especially lymphoma), severe drug-induced liver injury, exacerbation of congestive heart failure, and inflammatory CNS syndromes [12,13].

The risk of inflammatory central nervous system events such as ATM is increased in patients with RA treated with TNF inhibitors. Idiopathic transverse myelitis typically presents with acute or subacute onset of bilateral motor, sensory, and autonomic dysfunction below the level of the lesion, with a well-defined sensory level, paraparesis or quadriparesis, and sphincter dysfunction, such as that observed in our patient [14]. While idiopathic TM presents with symmetric motor and sensory deficits and more complete transverse involvement, MS-related myelitis typically presents as “partial” ATM, with asymmetric motor and sensory deficits and often incomplete transverse involvement of the cord. TNF-inhibitor-induced myelopathy shares many clinical features with idiopathic TM but has some distinguishing characteristics. These cases typically present with isolated spinal cord lesions, normal brain MRI at onset, and objective evidence of active inflammation (gadolinium enhancement and CSF pleocytosis or oligoclonal bands), whereas idiopathic TM often lacks gadolinium enhancement and oligoclonal bands are less frequently detected [13,14,15].

A systematic review and meta-analysis published in 2024 analyzed data from 1.1 million patients with autoimmune diseases who developed new-onset inflammatory CNS events after initiating TNF inhibitors. The incidence was estimated at 2.0 to 13.4 per 10,000 person-years. TNF inhibitor exposure was associated with a 36% increased risk of any inflammatory CNS disease compared to conventional therapies, with similar risk observed across different underlying autoimmune diseases and TNF inhibitor types, including etanercept [14,15,16]. A population-based study from Canada similarly found that among patients with rheumatic diseases, anti-TNF-α exposure was associated with a more than twofold increased risk of MS incidence [16].

In a retrospective cohort study, 65% of patients with CNS demyelinating events during TNF inhibitor exposure met the 2017 McDonald criteria for MS at the time of their initial neurological event, and 75% met the criteria by the end of follow-up. Over a median follow-up of 26 months, nearly half experienced clinical relapses, and two-thirds developed new MRI lesions, indicating a relapsing course consistent with MS in the majority of cases. Notably, the discontinuation of TNF inhibitors did not universally halt disease activity, and more than half of the patients demonstrated further evidence of CNS demyelination. This supports a causal relationship in some cases, although the demyelinating process may persist or evolve independently after drug discontinuation [17].

TNF-α was originally described as a circulating factor causing tumor necrosis and as a pro-inflammatory cytokine; however, subsequent research has shown that TNF-α exhibits a broad range of cellular effects, spanning the regulation of essential processes across multiple organ systems and the pathogenesis of autoimmune and inflammatory diseases. This functional diversity reflects its complex underlying biology. TNF-α is synthesized by multiple cell types, with macrophages and monocytes serving as the primary producers, particularly during acute inflammation [18]. TNF-α exists in both a transmembrane form (mTNF-α) and a soluble form (sTNF-α), which signal through two distinct receptors: TNFR1 and TNFR2. TNFR1 is ubiquitously expressed and activated by both molecular forms, mediating predominantly pro-inflammatory and pro-apoptotic effects. TNFR2 is expressed primarily on immune cells, endothelial cells, fibroblasts and subset of neurons, and is only fully activated in a soluble form, promoting neuroprotection, remyelination, and regulatory T-cell function [4,19].

Multiple TNF-α inhibitors (TNFIs) are approved for the treatment of a variety of inflammatory conditions, including rheumatoid arthritis (RA), psoriasis/psoriatic arthritis, ankylosing spondylitis, and inflammatory bowel disease. There are three types of TNFI: monoclonal antibodies that bind TNF-α (infliximab, adalimumab, and golimumab), a pegylated Fab’ fragment, certolizumab pegol, and a soluble TNF-α receptor, etanercept. There is some variation in the range of diseases that various TNFIs may treat, due to TNFI-related factors (different structures, mechanisms of immune modulation, pharmacokinetics, and immunogenicity) and disease-specific pathogenicity [18].

The use of TNFIs has been associated with a variety of side effects, including central and peripheral nervous system complications. Central nervous system (CNS) events include both demyelinating disorders (multiple sclerosis, optic neuritis, transverse myelitis, neuromyelitis optica spectrum disorder) and non-demyelinating conditions (neurosarcoidosis, meningoencephalitis, leptomeningitis, vasculitis). The mechanism of CNS complications remains poorly understood, although several mechanisms have been proposed:

1. Limited blood–brain barrier penetration of TNFIs. TNFIs are large macromolecules that cannot traverse the tight junctions of the brain capillary endothelium. This might result in an imbalance between peripheral, suppressed TNF-α and central TNF-α that remains active or dysregulated, promoting paradoxical CNS inflammation [6,19].
2. Upregulation of TNF expression. TNFI may sequester soluble TNF-α in the periphery, leading to the possible compensatory upregulation of TNF-α expression or TNF-α receptor signaling that could trigger an inflammatory cascade [4].
3. Inhibition of apoptosis. TNF-α has pro-apoptotic effects. TNFI may inhibit the apoptosis of autoreactive T cells, which may then enter the CNS and cause demyelination [4].
4. The induction of autoimmunity has also been proposed through the inhibition of TNF-mediated promotion of T-regulatory cell survival and proliferation [4].
5. Altered downstream cytokine responses. TNFI disrupts the normal cytokine network, leading to the dysregulation of type I interferons and interleukin-12, which may create a pro-inflammatory state in the CNS despite peripheral immunosuppression. [3,15].
6. Different effects of TNFR1 and TNFR2. TNFR1 mediates pro-inflammatory signaling and can lead to cell apoptosis, while TNFR2 modulates immune function, tissue preservation, and promotes oligodendrocyte differentiation and myelin repair. Non-selective TNF-α inhibition might remove neuroprotective TNFR2-mediated effects and may help explain why TNFI can worsen multiple sclerosis [19].

Several aspects of this case are noteworthy. TNF-inhibitor-associated demyelinating events typically improve or stabilize after drug discontinuation and high-dose corticosteroid therapy [14,15,16,17]. In contrast, our patient demonstrated a biphasic course with initial improvement followed by clinical worsening, an atypical trajectory in this context. The marked response to plasma exchange suggests a humoral inflammatory component rather than purely drug-induced demyelination, a pattern more characteristic of NMO/AQP4- or MOG-associated disease [14,15,16,17].

Additionally, the presence of a longitudinally extensive spinal cord lesion (T10–L1) is atypical for TNF-inhibitor-associated myelitis, which more commonly presents with shorter, MS-like partial transverse lesions. The extensive, centrally located lesion with cord expansion is more suggestive of antibody-mediated demyelination or an unusually severe inflammatory phenotype [1,2,3,4,5,14,15,16,17]. Isolated spinal cord involvement without brain lesions further distinguishes this case, as the largest case series of TNF-inhibitor-associated inflammatory events reported multifocal CNS involvement in 86% of patients [14].

On the other hand, the presence of CSF oligoclonal bands and elevated kappa free light chains in our case—findings more typical of MS and less frequently reported in TNF-inhibitor-associated disease—adds further complexity [14,15,16,17]. The combination of CSF-restricted oligoclonal bands (OCBs) and elevated KFLCs strongly favors a primary central nervous system demyelinating process, most likely MS rather than TNF-α-inhibitor-associated demyelination. OCBs are detected in greater than 90% of MS cases and reflect a chronic, compartmentalized intrathecal B-cell response that is the immunopathological hallmark of MS, while CSF-restricted OCB and elevated KFLC are typically absent or rare in purely drug-induced TNF-inhibitor-associated demyelination. It is important to note that OCBs are present in only approximately 20–30% of NMOSD cases and 10–20% of MOGAD cases, making these diagnoses less likely given the current CSF profile, though they cannot be excluded without serologic testing. Additionally, there are no well-defined differences in CSF profile between idiopathic MS and TNF alpha-induced demyelination. MOGAD can present with both short and longitudinally extensive lesions, but LETM is common, often involving the caudal spinal cord [20,21]. Differential diagnosis of LETM is illustrated in Table 1.

Taken together, the constellation of longitudinally extensive myelitis, steroid refractoriness requiring plasma exchange, biphasic progression, CSF oligoclonal bands, and isolated spinal involvement may represent either a severe phenotype of TNF-inhibitor-associated CNS inflammation or a previously unrecognized antibody-mediated disorder potentially triggered by TNF-inhibitor therapy. The temporal relationship to etanercept exposure (two years of therapy) is within the reported range for TNF-α-inhibitor-associated demyelination (median onset approximately 10–27 months after initiation) [4,10]. However, this temporal association alone does not establish causation, particularly when CSF biomarkers characteristic of MS is present. Hence, it remains plausible that etanercept unmasked or triggered a first clinical attack of MS in a genetically predisposed individual, rather than causing a de novo drug-induced demyelinating event, although repeated brain MRIs have remained normal. To our knowledge, reports of TNF-inhibitor-associated myelitis evolving into longitudinally extensive disease with steroid refractoriness and subsequent responsiveness to plasma exchange remain limited, suggesting that this case may expand the recognized clinical spectrum of TNF-α-related CNS inflammatory events.

Paraneoplastic etiologies of LETM were also considered, prompting a comprehensive evaluation for underlying malignancy. This workup included computed tomography (CT) of the chest, abdomen, and pelvis with and without intravenous contrast, carcinoembryonic antigen (CEA) testing, esophagogastroduodenoscopy (EGD), colonoscopy, and screening mammography. In addition, an extensive paraneoplastic antibody panel was performed. The following antibodies were tested and returned negative: amphiphysin, ANNA-1 (anti-Hu), CRMP5 (anti-CV2), KLHL11, PCA-1 (anti-Yo), and PCA-2. Additional antibodies associated with autoimmune and inflammatory central nervous system disorders were also evaluated and were negative, including anti-glial fibrillary acidic protein (GFAP), glutamic acid decarboxylase 65 (GAD65), and vimentin IgG.

Infectious etiologies were considered and excluded by extensive evaluation. At Mayo Clinic, the standard meningitis/encephalitis panel on CSF includes testing for the following: herpes simplex virus type 1 (HSV-1), herpes simplex virus type 2 (HSV-2), varicella-zoster virus (VZV), cytomegalovirus (CMV), Epstein–Barr virus (EBV), human herpesvirus 6 (HHV-6), enterovirus D68, enterovirus A71, poliovirus, coxsackievirus, echovirus, West Nile virus, Japanese encephalitis virus, Eastern equine encephalitis virus, Western equine encephalitis virus, St. Louis encephalitis virus, Zika virus, dengue virus, human immunodeficiency virus (HIV), human T-lymphotropic virus type 1 (HTLV-1), influenza A and B, measles virus, mumps virus, rubella virus, severe acute respiratory syndrome coronavirus 2 (SARS-CoV-2), rabies virus, *Staphylococcus aureus*, *Streptococcus* species, *Mycobacterium tuberculosis*, *Mycoplasma pneumoniae*, *Chlamydia pneumoniae*, *Legionella pneumophila*, *Treponema pallidum*, *Borrelia burgdorferi*, *Leptospira* species, *Schistosoma* species, *Toxoplasma gondii*, *Aspergillus* species, *Candida* species, *Cryptococcus neoformans*, *Coccidioides immitis*, *Histoplasma capsulatum*, and *Blastomyces dermatitidis*. Finally, particular attention was given to endemic and emerging pathogens relevant to northwestern Wisconsin. which were evaluated both in cerebrospinal fluid (CSF) and blood, including blood cultures and targeted testing for regionally prevalent tick-borne infections [22,23,24]. All results were negative, including studies for Lyme disease, babesiosis, anaplasmosis, and Powassan virus.

We have also considered an association with RA and considered that LETM might be manifestation of RA; however, LETM is not considered a true inflammatory manifestation of RA. In contrast to conditions such as systemic lupus erythematosus (SLE) or Sjögren’s syndrome, RA is not associated with the primary inflammatory involvement of the spinal cord parenchyma [25,26]. Instead, spinal cord dysfunction in RA is almost entirely attributable to compressive mechanisms, rather than direct immune-mediated inflammation of the central nervous system.

In cases like the one we have reported here, the positive OCBs and elevated KFLCs favored MS, and close clinical and radiographic follow-up with repeated brain MRI in 3–6 months was recommended to assess the possibility of the development of new lesions. Due to the longitudinally extensive lesions, NMOSD or MOGAD were still considered even with initially negative serologies, and repeated antibody testing is warranted [21]. Unfortunately, the patient was lost in follow-up as she travelled to continue treatment in another country, making the above information unavailable.

## 4. Conclusions

This case is notable for several atypical features, including a biphasic clinical course with initial steroid responsiveness followed by worsening, steroid refractoriness requiring plasma exchange, and a marked response to PLEX suggestive of a humoral inflammatory component. The presence of a longitudinally extensive, centrally located spinal cord lesion with cord expansion (T10–L1), isolated spinal involvement without brain lesions, and CSF oligoclonal bands with elevated kappa free light chains and mild pleocytosis further distinguishes this presentation from typical TNF-inhibitor-associated demyelination. Collectively, these findings raise concern for either an unusually severe phenotype of TNF-inhibitor-associated CNS inflammation or a previously unrecognized antibody-mediated demyelinating disorder potentially triggered by TNF-inhibitor therapy, warranting close clinical follow-up. Further research and systematic pharmacovigilance are needed to better define the neurologic risk profile of TNF-α therapy and to clarify long-term outcomes in affected patients.

## Figures and Tables

**Table 1:** The most common differential diagnoses of LETM.

	Clinical Course and Characteristics	MRI Findings	CSF Findings	Serology on Serum
LETM-idiopathic	• Acute or subacute • Can have relapses	• Centrally located T2 hyperintensity • Lesion length 3–4 segments • Focal peripheral gadolinium enhancement • Cord swelling	• Pleocytosis and OCB are variable	• Negative
MOGAD	• Acute • Young adults/children • Male-predominant	• Gray matter restriction (H sign) • Fluffy disorders • Frequently has absence of gadolinium enhancement	• Marked neutrophilic pleocytosis • OCB absent	• MOG Ig Ab • Unlike AQP4-IgG NMSOD other autoimmune antibodies are usually absent
MS associated ATM	• Females in 20–40 s • Acute or subacute onset • Variable relapses	• Short segment, wedge-shaped lesion	• Mild pleocytosis • OCB common	EBV antibodies
NMOSD	• Rapid nadir and relapsing course • The most common cause • 30% have an associated autoimmune disease	• Central cord involvement • “Bright spotty” lesions	• Pleocytosis common • OCB rare	• AQP4-IgG-positive • 50% have other Ab-positive (ANA), anti-Ro/SS-A antibodies, anti-thyroperoxidase antibodies, and anti-double-stranded DNA antibodies.
Paraneoplastic Myelitis	• Subacute and progressive. • May or may not have a formal diagnosis of a malignancy (lung, breast, lymphoma)	• Variable	• Pleocytosis with elevated protein • May have OCBs	• Paraneoplastic antibodies (anti-CRMP5/CV2, anti-amphiphysin, anti-GFAP, anti-Hu)
Neurosarcoidosis	• Subacute and typically progressive • Can be associated with constitutional symptoms	• Dorsal subpial linear enhancement (≥2 segments); “trident sign” on axial • Persistent enhancement >2 months	• Pleocytosis (lymphocytic • Elevated protein • Hypoglycorrhachia (11%) • Elevated ACE (18%)	• Serum ACE (low sensitivity) • Chest CT/PET-positive • Tissue biopsy confirmatory

Abbreviations: Ab—antibodies; ATM—acute transverse myelitis; CSF—cerebrospinal fluid; EBV—Epstein–Barr virus; LETM—longitudinally extensive transverse myelitis; MOG—myelin oligodendrocyte glycoprotein; MRI—magnetic resonance imaging; NMOSD—neuromyelitis optica spectrum disorder.

## Data Availability

The original contributions presented in this study are included in the article. Further inquiries can be directed to the corresponding author.

## References

[1] Tisavipat N., Flanagan E.P. (2023). Current perspectives on the diagnosis and management of acute transverse myelitis. Expert. Rev. Neurother..

[2] Lopez Chiriboga S., Flanagan E.P. (2021). Myelitis and Other Autoimmune Myelopathies. Contin. (Minneap. Minn.).

[3] Abbatemarco J.R., Galli J.R., Sweeney M.L., Carlson N.G., Samara V.C., Davis H., Rodenbeck S., Wong K.H., Paz Soldan M.M., Greenlee J.E. (2021). Modern Look at Transverse Myelitis and Inflammatory Myelopathy: Epidemiology of the National Veterans Health Administration Population. Neurol. Neuroimmunol. Neuroinflamm..

[4] Kemanetzoglou E., Andreadou E. (2017). CNS Demyelination with TNF-α Blockers. Curr. Neurol. Neurosci. Rep..

[5] Jonsson D.I., Sveinsson O., Moeini N., Pivac E., Wirdefeldt K., Brundin L., Iacobaeus E. (2025). Incidence, Etiology, and Long-Term Outcome of Acute Myelitis in Stockholm County, Sweden: A Population-Based Study. Neurol. Neuroimmunol. Neuroinflamm..

[6] Gritsch D., Valencia-Sanchez C. (2022). Drug-related immune-mediated myelopathies. Front. Neurol..

[7] Agmon-Levin N., Kivity S., Szyper-Kravitz M., Shoenfeld Y. (2009). Transverse myelitis and vaccines: A multi-analysis. Lupus.

[8] Chey S.Y., Kermode A.G. (2022). Central Nervous System Demyelination Related to Tumour Necrosis Factor Alpha Inhibitor. Mult. Scler. J. Exp. Transl. Clin..

[9] Valdés J.M., Roa B., Orellana P., Galleguillos L. (2025). Longitudinally extensive transverse myelitis associated with long-term adalimumab therapy: A diagnostic challenge. Clin. Neurol. Neurosurg..

[10] Barreras P., Mealy M.A., Pardo C.A. (2017). TNF-alpha inhibitor associated myelopathies: A neurological complication in patients with rheumatologic disorders. J. Neurol. Sci..

[11] Sukal S.A., Nadiminti L., Granstein R.D. (2006). Etanercept and demyelinating disease in a patient with psoriasis. J. Am. Acad. Dermatol..

[12] Zheng B., Liu M., Dai D., Shang Y., Dou X., Liu B., Zhong Z., Huang S., Luo D. (2024). Safety of TNF-α inhibitors: A real-world study based on the US FDA Adverse Event Reporting System Database. Medicine.

[13] Li M., You R., Su Y., Zhou H., Gong S. (2023). Characteristic analysis of adverse reactions of five anti-TNFɑ agents: A descriptive analysis from WHO-VigiAccess. Front. Pharmacol..

[14] Xie W., Sun Y., Zhang W., Zhu N., Xiao S. (2024). Risk of Inflammatory Central Nervous System Diseases After Tumor Necrosis Factor–Inhibitor Treatment for Autoimmune Diseases: A Systematic Review and Meta-Analysis. JAMA Neurol..

[15] Seror R., Richez C., Sordet C., Rist S., Gossec L., Direz G., Houvenagel E., Berthelot J.M., Pagnoux C., Dernis E. (2013). Pattern of demyelination occurring during anti-TNF-α therapy: A French national survey. Rheumatology.

[16] Li L., Aviña-Zubieta J.A., Bernstein C.N., Kaplan G.G., Tremlett H., Xie H., Peña-Sánchez J.N., Marrie R.A., Etminan M. (2023). Risk of Multiple Sclerosis Among Users of Antitumor Necrosis Factor α in 4 Canadian Provinces: A Population-Based Study. Neurology.

[17] Hutto S.K., Rice D.R., Mateen F.J. (2021). CNS demyelination with TNFα inhibitor exposure: A retrospective cohort study. J. Neuroimmunol..

[18] Gough P., Myles I.A. (2020). Tumor Necrosis Factor Receptors: Pleiotropic Signaling Complexes and Their Differential Effects. Front. Immunol..

[19] Kunchok A., Aksamit AJJr Davis JM3rd Kantarci O.H., Keegan B.M., Pittock S.J., Weinshenker B.G., McKeon A. (2020). Association Between Tumor Necrosis Factor Inhibitor Exposure and Inflammatory Central Nervous System Events. JAMA Neurol..

[20] Carta S., Ferraro D., Ferrari S., Briani C., Mariotto S. (2022). Oligoclonal bands: Clinical utility and interpretation cues. Crit. Rev. Clin. Lab. Sci..

[21] Kalnins A., Lewis L.M., Soderlund K.A., Austin M.J., Chu S., Hawley D.B., Kontzialis M., Levy M., McMenamy J., Expert Panel on Neurologic Imaging (2026). ACR Appropriateness Criteria^®^ Demyelinating Diseases. J. Am. Coll. Radiol..

[22] Dumic I., Vitorovic D., Spritzer S., Sviggum E., Patel J., Ramanan P. (2018). Acute transverse myelitis-A rare clinical manifestation of Lyme neuroborreliosis. IDCases.

[23] Dumic I., Glomski B., Patel J., Nordin T., Nordstrom C.W., Sprecher L.J., Niendorf E., Singh A., Simeunovic K., Subramanian A. (2021). “Double Trouble”: Severe Meningoencephalitis Due to Borrelia burgdorferi and Powassan Virus Co-Infection Successfully Treated with Intravenous Immunoglobulin. Am. J. Case Rep..

[24] Cosiquien R.J.S., Stojiljkovic N., Nordstrom C.W., Amadi E., Lutwick L., Dumic I. (2023). *Anaplasma phagocytophilum* Encephalitis: A Case Report and Literature Review of Neurologic Manifestations of Anaplasmosis. Infect. Dis. Rep..

[25] DeQuattro K., Imboden J.B. (2017). Neurologic Manifestations of Rheumatoid Arthritis. Rheum. Dis. Clin. N. Am..

[26] Flanagan E.P., Kaufmann T.J., Krecke K.N., Aksamit A.J., Pittock S.J., Keegan B.M., Giannini C., Weinshenker B.G. (2016). Discriminating Long Myelitis of Neuromyelitis Optica From Sarcoidosis. Ann. Neurol..

